# “Breath figures” on leaf surfaces—formation and effects of microscopic leaf wetness

**DOI:** 10.3389/fpls.2013.00422

**Published:** 2013-10-24

**Authors:** Juergen Burkhardt, Mauricio Hunsche

**Affiliations:** ^1^Plant Nutrition Group, Institute of Crop Science and Resource Conservation, University of Bonn, Bonn, Germany; ^2^Horticultural Science Group, Institute of Crop Science and Resource Conservation, University of Bonn, Bonn, Germany

**Keywords:** cloud condensation nuclei, deliquescence, dew, foliar nutrition, Hofmeister series, leaf boundary layer, plant-atmosphere interaction, surface tension

## Abstract

“Microscopic leaf wetness” means minute amounts of persistent liquid water on leaf surfaces which are invisible to the naked eye. The water is mainly maintained by transpired water vapor condensing onto the leaf surface and to attached leaf surface particles. With an estimated average thickness of less than 1 μm, microscopic leaf wetness is about two orders of magnitude thinner than morning dewfall. The most important physical processes which reduce the saturation vapor pressure and promote condensation are cuticular absorption and the deliquescence of hygroscopic leaf surface particles. Deliquescent salts form highly concentrated solutions. Depending on the type and concentration of the dissolved ions, the physicochemical properties of microscopic leaf wetness can be considerably different from those of pure water. Microscopic leaf wetness can form continuous thin layers on hydrophobic leaf surfaces and in specific cases can act similar to surfactants, enabling a strong potential influence on the foliar exchange of ions. Microscopic leaf wetness can also enhance the dissolution, the emission, and the reaction of specific atmospheric trace gases e.g., ammonia, SO_2_, or ozone, leading to a strong potential role for microscopic leaf wetness in plant/atmosphere interaction. Due to its difficult detection, there is little knowledge about the occurrence and the properties of microscopic leaf wetness. However, based on the existing evidence and on physicochemical reasoning it can be hypothesized that microscopic leaf wetness occurs on almost any plant worldwide and often permanently, and that it significantly influences the exchange processes of the leaf surface with its neighboring compartments, i.e., the plant interior and the atmosphere. The omission of microscopic water in general leaf wetness concepts has caused far-reaching, misleading conclusions in the past.

## Introduction

“Breath figures” is a term used in material science to describe the condensation as well as the linked wetting and dewetting processes on different kinds of surfaces (Blaschke et al., 2012). The examination of breath figures has then been used as a method to characterize the degree of contamination on an otherwise homogenous surface (Kumar and Whitesides, 1994). The term was originally introduced by Aitken (1911) who noticed that water from exhaled breath condensing to clean glass surfaces was clearly visible as separate droplets. If the glass was contaminated with fine particles, however, the condensation would be strong but not visible, due to the formation of thin water films (Aitken, 1911). Condensation to deposited particles (“contaminants”) is also considered an essential factor in corrosion, and according to ISO 9223 wetting happens at 80% RH and above due to particle hygroscopicity (Schindelholz and Kelly, 2012).

In plant science, the influence of particles on condensation has not been considered sufficiently so far. On leaf surfaces, the commonly known form of condensation is morning dewfall. It develops during clear, calm nights, when plant surfaces cool down by radiational heat loss, and the surface temperature eventually reaches the dew point of the surrounding air. According to this common meteorological definition, dew formation thus starts when 100% relative humidity (RH) is reached at the actual leaf surface temperature, which normally means about 90% RH of the surrounding air (Monteith, 1957). It is usually neglected that the initiation of condensation on leaf surfaces likely starts on condensation nuclei, analogously to atmospheric cloud formation (Beysens, 1995). These nuclei are tiny hygroscopic particles, which are present on all kinds of leaf surfaces. They result from atmospheric dry deposition of aerosols or residues from evaporated rain droplets, while removal by rain is never complete (Neinhuis and Barthlott, 1998; Freer-Smith et al., 2005). Almost all aerosols are (partly) hygroscopic (Pöschl, 2005) and therefore cause a local reduction of the saturation vapor pressure. Even the commonly used expression “dry deposition” for aerosols is usually misleading, because many of the deposited substances become deliquescent at higher humidities (e.g., 75% RH for a NaCl particle). Equilibration with the surrounding RH happens very quickly (Pilinis et al., 1989) and many particles will therefore reach a transpiring leaf surface in deliquescent form.

Neglecting particle deliquescence can cause misleading conclusions. An example is the “wax degradation” phenomenon that was frequently found on conifer needles which were affected by air pollution caused forest decline. The phenomenon was intensively investigated in the 1980s and 1990s, but the investigations concentrated on the chemical composition of the waxes and could not explain the development of the phenomenon. However, the characteristic, amorphous appearance of epicuticular waxes can also be produced in a simple way by deliquescent particles covering the structures of the epicuticular waxes. This alternative explanation was suggested recently (Burkhardt, 2010) and its capability to explain the phenomenon was meanwhile demonstrated by experiment (Burkhardt and Pariyar, 2013). Because the minimum epidermal conductance g_min_, a key factor of tree drought tolerance, was also reduced by salt particles, and given the fact that particle accumulation on conifers can reach the amount of leaf waxes (up to more than 50 μg cm^−2^, Saebo et al., 2012), a direct link between particulate air pollution and drought symptoms of conifers might exist, with “wax degradation” as an indication of particle load (Burkhardt and Pariyar, 2013).

The second neglected factor for the formation of leaf wetness is foliar (mainly stomatal) transpiration. In the common definition of dewfall, the main source of water vapor for dew formation on plants is the surrounding atmosphere, with an eventual contribution by “distillation” from the soil (Monteith, 1957). On leaf surfaces, however, foliar transpiration is an additional water vapor source. The leaf boundary layer is humidified by this water vapor, leading to high water vapor concentration especially at the leaf surface (Schuepp, 1993; Roth-Nebelsick, 2007), which together with hygroscopic substances will lead to the formation of microscopic leaf wetness (Burkhardt and Eiden, 1994; Burkhardt et al., 1999). Although this process only involves small amounts of water, it might considerably change the transport between the leaf surface and the neighboring compartments, which is supported by the dependence of trace gas deposition on RH: for easily soluble compounds like NH_3_ and SO_2_, increasing trace gas deposition to cuticular surfaces (“non-stomatal fluxes”) was already found for 70% RH (van Hove et al., 1989; Burkhardt and Eiden, 1994; Wichink Kruit et al., 2008). The trace gas deposition to microscopic leaf wetness is also dependent on the chemical composition of the water, e.g., on pH or on leached manganese ions catalyzing SO_2_ oxidation (Burkhardt and Drechsel, 1997). Non-stomatal deposition is also significant for ozone, making up between 1/3 and 2/3 of total deposition (Coyle et al., 2009; Fowler et al., 2009; Launiainen et al., 2013). A positive relation of ozone deposition with RH was also found (Pleijel et al., 1995; Altimir et al., 2006. Lamaud et al., 2009).

Foliar fertilization is a complicated process with foliar uptake being the first decisive step (Fernandez and Brown, 2013). Continuing microscopic leaf wetness might contribute considerably to the foliar exchange of ions. When dilute solutions are applied, the highest uptake rates into leaves occur during the drying phase, presumably as a consequence of increasing concentrations (Eichert and Burkhardt, 2001). The high concentrations of electrolytes in deliquescent particles are expected to promote the gradient dependent exchange process across the leaf surface, and maintenance of high concentrations would therefore lead to high transport rates.

Macroscopic leaf wetness, i.e., visible wetting of leaves, usually has a large influence on the phyllosphere. For phyllospheric organisms, water is a key issue to survive (Beattie, 2011; Vorholt, 2012). The amount of water needed depends on the organism but usually “free water” (probably meaning visible water) is required by phyllospheric organisms like fungi, bacteria or insects and thus fosters phyllospheric life including plant pathogens (Huber and Gillespie, 1992). Microscopic leaf wetness might also influence the phyllosphere to a certain degree, but cannot be treated here in depth.

The aim of this contribution is to elucidate the mechanisms and conditions by which microscopic leaf wetness is formed and maintained. So far there have only been isolated reports and phenomenological descriptions, while an integrated view and a general concept detailing the occurrence and the functions of microscopic liquid water at the plant/atmosphere interface is missing.

## Detection of microscopic leaf wetness

**Figure 1:**
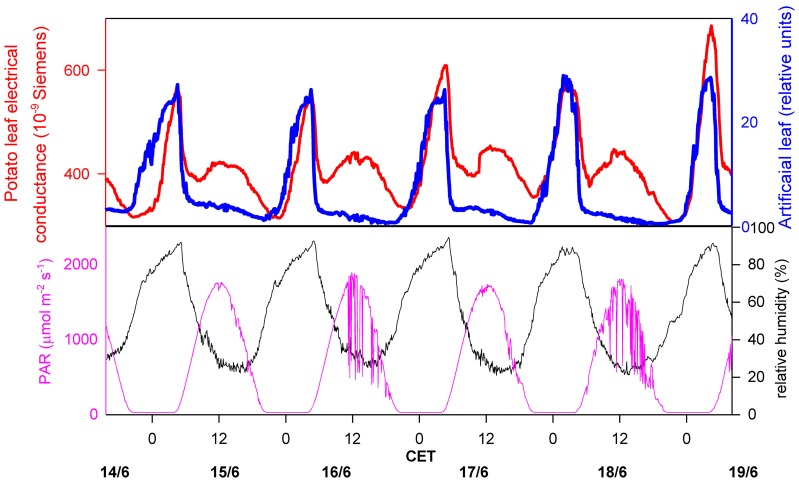
**Measurement of leaf wetness on a potato field, comparing an artificial leaf (blue line; Campbell leaf wetness sensor 237, Campbell Scientific, Logan, UT, USA), and a leaf wetness sensor directly attached to a potato leaf (red line, upper image; construction see Burkhardt and Gerchau, 1994).** Ambient air humidity (black line, lower picture) and photosyntheticcally active radiation (pink line, lower picture) are also shown. CET, Central European Time.

The most common method to determine (macroscopic) leaf wetness duration is the electrical resistance measurement of artificial leaves. A continuous resistance signal is produced, which is divided into “wet” or “dry” by defining a resistance threshold, based on the visual observation of wetness (Gillespie and Kidd, 1978; Fuentes and Gillespie, 1992; Huber and Gillespie, 1992; Armstrong et al., 1993; Sentelhas et al., 2007). For the detection of microscopic leaf wetness, a similar electronic device can be used, but the sensors to measure the electric resistance are directly attached to the leaf surface (Burkhardt and Gerchau, 1994). The signal is then compared to ambient RH (Burkhardt and Eiden, 1994), or to the signal of a commercial leaf wetness sensor, i.e., an artificial leaf. An example for the latter procedure is shown in Figure 1. The electrical conductance on potato leaves was measured in Southern Germany during a hot summer week, and was compared to the continuous signal of an artificial leaf sensor (237 Leaf Wetness Sensing Grid, Campbell Scientific, Logan, UT, USA) which was installed in close proximity. Photosynthetically active radiation (PAR) and ambient RH data were obtained from a weather station on the same field. For both wetness sensors, the nighttime increase is clearly visible and goes parallel with each other, with a significant decrease of resistance starting at about 60 to 70% RH of the surrounding air. During daytime, a different course of the signals is observed, with the sensor on the potato leaves showing a regular increase in the mornings, which is missing on the artificial leaf.

Because the leaf wetness signal is highly correlated with PAR, it is most probably the consequence of changing stomatal conductance, where transpired water coming from the stomata re-condenses on the leaf surface. This interpretation is supported by the results of a detailed study under completely controlled conditions using the same type of leaf wetness sensors on bean leaves. Under constant humidity and by changing light or changing CO_2_ concentration was the electrical leaf surface conductance closely correlated with stomatal conductance (Burkhardt et al., 1999). These results indicate that microscopic water can exist on leaf surfaces for extended times, even under hot, dry summertime conditions, and that the liquid water therefore is in an equilibrium state, reacting quickly to increased transpiration by the formation of more liquid water, and by a reduction of the water amount when the stomata close. This phenomenon can be explained by two processes. One is the leaf transpiration which creates a humid leaf boundary layer (LBL) including the proper leaf surface. During times of open stomata, leaf surface humidity (RHs) will mostly be determined by transpiration, with only limited influence by ambient RH. The distribution of leaf surface humidity is heterogeneous and will especially be high near to stomata (Schuepp, 1993; Roth-Nebelsick, 2007).The second process is a local reduction of the saturation vapor pressure by effects of the leaf surface material (sorption by the cuticle, deliquescence of hygrocopic leaf surface particles), or geometry (capillary condensation), which will be discussed in more detail within the next section. As also calculated in the next section, the hypothetical homogenous thickness of the liquid water is less than 1 μm. This small amount of water is not visible and is two orders of magnitude smaller than normal morning dewfall of up to 0.5 mm (Monteith, 1957). Although microscopic leaf wetness could be interpreted as a specific form of dewfall, meteorological instruments are not sensitive enough to detect and to filter it from other signals, neither by lysimeters for the amount of water, nor by flux measurements for the contribution to the energy budget.

So far, no field measurement techniques are known other than the indirect method where the signals from leaf wetness sensors are compared to ambient RH or to the signals from artificial leaf wetness sensors. Microscopic leaf wetness is also not visible without the use of microscopic techniques. While a combination of a gas exchange cuvette with a light microscope enabled the observation of microscopic water formed by stomatal transpiration and showed the influence of the leaf boundary layer (Burkhardt et al., 2001), the resolution of a light microscope is not high enough to study the interactions between leaf surface particles and stomatal transpiration. Detailed observations are enabled by environmental scanning electron microscopy (ESEM), where it is possible to study condensation processes at high resolution and under controlled humidity. A limitation of the ESEM technique to keep in mind is the fact that leaves are abscised and are not transpiring anymore, so RH and RHs are only regulated from outside. Another difficulty is the exact detection of leaf surface temperature in case thicker leaves or needles are used, because the necessary cooling happens from a small table below the sample. The ESEM observations are usually done at low temperatures of 2 to 5°C in order to reduce the necessary amount of water vapor molecules to reach high RH, which in most cases is not a limitation. ESEM observations have been used to study both condensation on ambient, untreated leaves and the changes resulting from changes in RH after spraying leaves with different types of solutions or dry aerosols (Burkhardt et al., 2012; Burkhardt and Pariyar, 2013).

## Processes leading to microscopic leaf wetness

The formation of microscopic leaf wetness as a reaction to increasing RHs likely is the result of one or several of five water-solid interaction processes (Mauer and Taylor, 2010): (1) adsorption to the leaf surface (cuticle, trichomes, particles), (2) uptake into the leaf surface by absorption, (3) uptake and formation of crystal hydrate, (4) capillary condensation at contact points or in pores in the form of menisci, or (5) deliquescence of hygroscopic material. In all cases, the formation of liquid water is energetically favored at a given RH and liquid water is formed at RH <100%. In order to decide which processes are involved in the formation of microscopic leaf wetness, different criteria can be used which are an estimation of the amount of water, the time of equilibration, and possibly also hysteresis effects, i.e., the quantity of liquid water is different when RH rises than it is when RH decreases.

The formation of crystal hydrate is not of importance here, at it is applicable only for specific salts and can be excluded for cuticles. The process of adsorption is a hysteresis free and physically well described process with RH-dependent exponential increase, but can only explain a few nanometers of liquid water on the respective surface. Capillary condensation occurs in pores, or where contact points between solids allow the formation of menisci (e.g., Eiden et al., 1994), and hysteresis may occur for this process, but the amount of water involved is small. The remaining processes are the cuticular absorption of water and the deliquescence of hygroscopic leaf surface particles. Both processes can attract enough water in an unsaturated atmosphere to explain the observations. It was suggested that cuticular water absorption happens mainly by polysaccharides (Dominguez et al., 2011). Water increases the viscous component of the cuticle, modifies its macroscopic appearance and affects its biomechanical properties, acting as a plasticizer (Dominguez et al., 2011). Cuticular water absorption could account for a water layer thickness of 0.05–2.5 μm [calculation based on cuticular thickness 4–30 μm, cuticular area weight 5–30 × 10^−3^ kg m^−2^; 1–8% water absorption; Chamel et al., 1991], while for trichomes the sorption can be higher (Fernandez et al., 2011).

The other process is the deliquescence of leaf surface particles. Leaf surface particles are mainly coming from atmospheric aerosol deposition. Aerosol particles are omnipresent, with characteristic aerosol number concentrations for particles <2.5 μm diameter of 4 (alpine), 10 (rural), and 20 (urban) μ g m^−3^ in Central Europe (Pöschl, 2005). The concentrations of condensation nuclei in continental air are one to two orders of magnitude higher than natural concentrations (Rosenfeld et al., 2008). Plant surfaces are a major terrestrial sink with considerable, plant species specific particle accumulation of up to 50 μg cm^−2^ and occasionally more (Burkhardt, 2010; Saebo et al., 2012; Popek et al., 2013). A large fraction of aerosols is hygroscopic and may act as cloud condensation nuclei (CCN) in the atmosphere (e.g., Pöschl, 2005). Salt particles (and some organic material like e.g., urea) are hygroscopic and become deliquescent at a defined RH DRH [e.g., ~75% for NaCl, 40% for NH_4_HSO_4_, and 80% for (NH_4_)_2_SO_4_], which equals the equilibrium vapor pressure above a saturated solution of the salt. Deliquescence is the dissolution of the salt particle in the water vapor of the surrounding air, which occurs when the vapor pressure of the surrounding air equals or exceeds DRH. The salts absorb exponentially more water with further increasing humidity (Pilinis et al., 1989; Zhao et al., 2008; Mauer and Taylor, 2010). This mechanism is similar to the activation of cloud condensation nuclei, although DRH is slightly different for deposited particles (Gao et al., 2007). With decreasing humidity usually hysteresis between DRH and crystallization (efflorescence) is observed, which is due to an energy barrier associated with nucleation of the solid during drying. The importance of particle deliquescence for plants became clear with the first detection of microscopic leaf wetness on spruce needles even during hot summer days and the subsequent identification of deposited aerosols as the main reason (Burkhardt and Eiden, 1994). However, “point of deliquescence” (DOP) describes the same phenomenon, as it was re-introduced by (Schönherr, 2001).

The amount of water attached to hygroscopic particles can be calculated, based on data of particle loading of leaf surfaces. Assuming a particle loading of 5 μg cm^−2^ ammonium sulfate (AMS) with DRH 80% RH and a subsequent humidity increase to 92% RH, the radii of the particles (which are assumed to be round) would have doubled (Tang et al., 1981) and the consequent 8-fold volume would result in a loading of 40 μg cm^−2^ AMS solution or 0.4 μm hypothetical homogeneous water film thickness. It is thus in a similar range as the cuticular water absorption capacity and also similar to the “effective water volume” of a few μm thickness calculated from measurements of ammonia absorption by (Chamel et al., 1991; van Hove and Adema, 1996). Thus, both the cuticular absorption of water and particle deliquescence may attract similar amounts of water vapor and could be responsible for the signal observed in Figure 1. The equilibration process of the salt solutions with RH and deliquescence, however, are considerably faster (in the range of milliseconds; Pilinis et al., 1989) than for cuticular sorption (in the range of several seconds; Chamel et al., 1991). It is likely that all four processes contribute to microscopic leaf wetness, with adsorption and capillary condensation as initial processes and subsequent attraction of larger amounts of water by absorption and deliquescence, and with immediate condensation and evaporation from particles and slower adjustment of the cuticular water content in response to changes in RHs.

Most leaf surfaces are hydrophobic, which is a result of both the surface chemistry and the microstructure of the surface (Holloway, 1969; Aryal and Neuner, 2010; Khayet and Fernandez, 2012; Rosado and Holder, 2013). Leaf surface hydrophobicity affects all processes of water formation except deliquescence, which only depends on the hygroscopicity of the particle. However, the shape of the water formed by a deliquescent particle will be influenced by leaf surface hydrophobicity. In addition, the final shape will also be influenced by ion specific effects and by the “history” of the surface (Burkhardt et al., 2012). For deliquescent NaCl particles on hydrophobic tomato cuticles, single droplets were formed repeatedly in repeated drying/wetting cycles observed in the ESEM. However, with the fifth cycle, thin crystals spread out in dendritic form on the surface (Burkhardt et al., 2012), a process showing the influence of surface “history.” Because microscopic leaf wetness is maintained by stomatal transpiration for longer times, small RHs changes will lead to repeated increase and decrease of ion concentrations and eventually to repeated efflorescence and deliquescence, enabling dynamic changes. All these processes will create an ageing process of the leaf surface. On a macroscopic level, ageing is usually related with decreasing contact angles (Cape, 1983; Boyce et al., 1991; van Wittenberghe et al., 2012).

## Physicochemical properties of microscopic leaf wetness

Hygroscopic leaf surface particles contribute to a reduction of the original cuticular hydrophobicity, and the microscopic leaf wetness formed by deliquescent particles results in highly concentrated solutions which have different properties compared to pure water. Physical effects include capillary condensation, capillary transport of substances, Marangoni flow (cyclic inward or outward movement within the droplet), the accumulation of dispersed substances at the edges (coffee-rings), the reduction of contact angles by preferential evaporation from droplet edges, and “line-pinning” of droplets during evaporation (Eiden et al., 1994; Deegan et al., 1997; Herminghaus et al., 2008; Xu et al., 2010; Hunsche and Noga, 2012). In addition, the ion concentrations within microscopic leaf wetness will often reach values >1 M, [saturated conditions at DRH, i.e., 6.1 M for NaCl, 8.6 M for NaClO_3_, 9.0 M for NH_4_HSO_4_, 5.7 M for (NH_4_)_2_SO_4_ at 20°C, respectively; (IFA, 2012)]. For concentrations >0.1 M, ion-specific properties become important (Lo Nostro and Ninham, 2012); these include viscosity, surface tension, the “hydrophobic effect,” and salting-in/salting-out, the latter describing the solubility of non-electrolytes in electrolyte solution compared to pure water. Water surface tension is the most important physicochemical parameter in this context. It reflects the dispersive forces across the phase boundary as well as the specific forces within one phase such as hydrogen bonding (Dutcher et al., 2010). Water surface tension changes in a concentration-dependent and ion-specific manner at high ionic concentrations, which is related to the respective distribution of the ions between the surface and the bulk of a water droplet and follows the order of the Hofmeister (or lyotropic) series (Collins and Washabaugh, 1985; Bostrom et al., 2001; Pegram and Record, 2007; Liao et al., 2009; dos Santos et al., 2010; Dutcher et al., 2010; Zhang and Cremer, 2010). There is a series for anions and for cations, respectively, but anions have a stronger effect than cations. For anions, the Hofmeister series is

$${\text{SO}}_{4}^{2-}>{\text{F}}^{-}>{\text{Cl}}^{-}>{\text{Br}}^{-}>{\text{NO}}_{3}^{-}>{\text{ClO}}_{3}^{-}>{\text{I}}^{-}>{\text{ClO}}_{4}^{-}>{\text{SCN}}^{-}.$$

Ions that are considered kosmotropic are on the left side of the series and chaotropic ions are on the right. Thus, the sulfate anion, which is on the kosmotropic side of the series, decreases the solubility of non-polar molecules, increases the hydrophobic interaction (“salting out”), and increases surface tension, whereas the iodide and the thiocyanate ion both belong to the chaotropic ions, which increase the solubility of non-polar molecules, weaken the hydrophobic interaction (“salting in”), and decrease surface tension.

Together with cuticular hydrophobicity and stomatal geometry, water surface tension was the central argument of Schönherr and Bukovac (1972) for excluding any stomatal uptake of water or solutes. According to their investigations, a surface tension <30 mN m^−1^ would be needed for water to enter into stomata, which with the exception of organosilicons (Stevens, 1993) cannot be reached with most surfactants. The surface tension of pure water droplets is 72 mN m^−1^ at 25°C. For saturated chaotropic NaClO_3_ solutions (concentration 7 M), a surface tension of ~50 mN m^−1^ was reached (Burkhardt et al., 2012). Although the surface tension was still higher than the 30 mN m^−1^ and therefore no stomatal penetration should have happened, the reaction of apple leaves to the application of NaClO_3_ droplets surfactant indicated that stomatal uptake had taken place; even the addition of an organosilicon surfactant did not cause a stronger reaction (Burkhardt et al., 2012).

Following the first successful experimental proof of stomatal penetration with the use of nanoparticles (Eichert et al., 2008; Fernandez and Eichert, 2009), that NaClO_3_ experiment further supported the occurence of stomatal penetration by solutes and in addition provided an explanation why the long lasting paradigm that had excluded stomatal uptake was mistaken: the reasoning of Schönherr and Bukovac (1972) had been based on the water surface tension of pure water, while deliquescent particles may form thin, mobile solutions with low surface tension. In addition, the formation of thin films by deliquescent particles is not consistent with another essential precondition for the argument of Schönherr and Bukovac (1972), which acts on an assumption where the stomatal opening is completely covered by a droplet.

## The importance of microscopic leaf wetness: hypotheses and discussion

Based on the existing knowledge about microscopic leaf wetness and its development, it can be assumed that such minute amounts of liquid water exist on almost any plant to a certain degree, and in many cases almost permanently. The major reason for this hypothesis is the fact that hygroscopic particles are ubiquitous and will start to deposit immediately after unfolding of a leaf. Stomatal transpiration will inevitably increase RHs at least in the surroundings of the stomata above a value of 75%, which is the DRH of most common atmospheric aerosols. Plants in very dry regions, especially with CAM photosynthesis might represent an exception during daytime. In many cases, however, permanent microscopic leaf wetness might result from the fact that RHs exceeds 75% during daytime due to stomatal transpiration, and during nighttime due to high ambient RH.

As a second, related hypothesis, it can be assumed that with increasing age an increasing number of liquid water connections into the stomata will develop. The formation process of this “hydraulic activation of stomata” (HAS) affects individual stomata: the hydrophobic cuticle lining the stomatal walls has to become covered by a thin liquid water layer (Burkhardt, 2010). This process is favored by hygroscopic particles. Air pollution is expected to produce a high degree of HAS due to high particle deposition. It is also hypothesized that solutions containing any surfactants, but also concentrated solutions of chaotropic salts will be specifically efficient in creating HAS.

For a first experimental approach to test the last hypothesis, solutions (50 mM) of two chaotropic ions (KI, KSCN) were sprayed on Scots pine (*Pinus sylvestris*) needles, and the needles were observed the following day under changing RH by ESEM. The instrumental conditions and procedures for the ESEM were the same as used before (Burkhardt and Pariyar, 2013). The outcome of this experiment is demonstrated by two movies.

It is important to note that both movies do not show transpiration effects, as needles were abscised and were within the vacuum chamber of the ESEM. RH was only manipulated from outside.

It also has to be noted that the “stomatal openings” only show the entrance to the epistomatal chamber of the pine needles. The guard cells are located at the bottom of this opening and cannot be seen. Nevertheless, regarding the geometrical situation of interest, the epistomatal chamber has the same features as an open stoma, i.e., a diverging and a converging portion. This makes it comparable to the geometrical situation used by (Schönherr and Bukovac, 1972) to derive their conclusion that water uptake into the stomata is impossible.

In both movies, the strong dynamics of deliquescence can be seen. Movie 1 shows the repeated deliquescence and efflorescence of KI. The efflorescence of the KI crystals is highly unpredictable and repeatedly the crystallization takes place within the epistomatal chambers, a clear indication that KI solution had entered there. The movement of the solution into epistomatal chambers can be seen even clearer in Movie 2. Here, KSCN was used because it is on the far chaotropic side of the Hofmeister series. The movie follows one deliquescence process of KSCN. The solution shows an extremely flat contact angle, and it is clearly recognizable that the deliquescent KSCN solution enters the epistomatal chamber. Both movies can thus be taken as additional proofs for the stomatal uptake of aqueous solutions. They can also be interpreted as a first successful support for the hypothesis that chaotropic salts are more easily penetrating into the stomata. Finally, they can be taken as a confirmation of Aitken's observation of “breath figures,” i.e., the water vapor condenses to a “contaminant” on a hydrophobic surface, consequently forms liquid water in a flat, non-droplet like shape, and spreads out easily.

## Conclusions and recommendations

Microscopic leaf wetness can play an important role for trace gas deposition and for ion fluxes across the plant surface. Increased ammonia deposition over a Douglas fir forest was observed above 70% RH at night and even lower at daytime (Wyers and Erisman, 1998), and over a grassland above 71% RH (Wichink Kruit et al., 2008). During daytime, a contribution of 66% to 88% was found for “cuticular ammonia deposition” to a maize canopy (Walker et al., 2013). For ammonia, this microscopic leaf wetness will enable bi-directional “cuticular” gaseous exchange, depending on dynamic environmental conditions and the compensation point (Flechard et al., 1999; Burkhardt et al., 2009; Sutton et al., 2009). Non-stomatal ozone deposition is more difficult to explain, as ozone is less soluble than ammonia, and no obvious chemical reactions can account for the observed non-stomatal losses. However, several reaction mechanisms of ozone with atmospheric aerosols have been discussed (Oum et al., 1998; Jacob, 2000; Roeselova et al., 2003), and although such mechanisms have so far been out of focus in the search for reasons explaining non-stomatal ozone deposition, they should be considered taking into account the likely continuing occurrence of highly concentrated solutions on leaf surfaces.

Microscopic leaf wetness influences plant physiology. Leaf surface particles increase HAS, and the liquid water connections formed between the leaf surface and the apoplast along the stomatal walls have an influence on water and nutrient fluxes. Increased transpiration and reduced water use efficiency caused by leaf surface particles were observed for particle exclusion (Pariyar et al., 2013) as well as for particle amendment (Burkhardt et al., 2001). The stomatal uptake of nutrients is enabled as well as the stomatal leaching of ions, although an experimental proof for the latter is still missing. Sound reasons for nocturnal transpiration (Caird et al., 2007) have so far been missing, and nocturnal stomatal nutrient uptake might represent one benefit for the plant.

The development of models addressing both the physical mechanisms as well as the (physico)chemistry of microscopic leaf wetness would be useful. So far, morning dewfall is considered a micrometeorological phenomenon and is assessed via a negative energy balance. In order to address the relevance of the mechanism, the implementation of microphysical aerosol models would be useful, introducing “DCN” (dew condensation nuclei) on leaf surfaces, with a similar formalism as atmospheric CCN. For this purpose, advanced chemical aerosol models could be introduced into models of plant-atmosphere interaction.

The influence of deposited aerosols on plant physiology and on plant-atmosphere interactions has so far been neglected in plant science as well as in micrometeorology. Leaf surface particles were assumed to stay chemically inert. Leaf surface wetness was defined by visible detection and was considered to exist as pure water or strongly dilute solutions. Microscopic leaf wetness develops by the hygroscopic action of fine particles, with water vapor mainly from stomatal transpiration. “Breath figures” on leaf surfaces are microscopically thin films as well as droplets, which are highly dynamic in concentration and extension. They interact with the atmosphere by bi-directional gas fluxes and with the apoplast via HAS by hydraulic signals and the exchange of aqueous solutions. The consideration of these processes in broadened concepts of plant-atmosphere interactions is highly desirable. Including existing aerosol models into leaf surface exchange models seems a priority task on this road.

## Conflict of interest statement

The authors declare that the research was conducted in the absence of any commercial or financial relationships that could be construed as a potential conflict of interest.

## Supplementary material

Movie 1 **Potassium iodide (KI) crystals on a *Pinus sylvestris* needle under changing humidity in an environmental scanning electron microscope.** Three deliquescence/efflorescence cycles are shown, cycling between approximately 55 and 70% RH.

Movie 2 **Potassium thyocyanate (KSCN) crystals on a *Pinus sylvestris* needle under increasing humidity in an environmental scanning electron microscope.** Humidity increases from 60 to 65% RH.

## Abbreviations

AMS: ammonium sulfate
CET: Central European Time
DRH: deliquescence relative humidity
ESEM: environmental scanning electron microscopy
HAS: hydraulic activation of stomata
LBL: Leaf boundary layer
PAR: photosynthetically active radiation
RH: relative humidity
RHs: relative humidity at the leaf surface
SEM: scanning electron microscopy.
